# Supplementary material for: The impact of autophagy on arbovirus infection of mosquito cells
Source: PLoS Negl Trop Dis. 2020 May 18;14(5):e0007754. doi: 10.1371/journal.pntd.0007754 (PMC7259790; doi:10.1371/journal.pntd.0007754)
Supplement: S1 Supporting Information — (DOCX) [file pntd.0007754.s001.docx]

**SUPPLEMENTAL INFORMATION**

**Viruses**

The ZIKV strain used in these studies was PRVABC 59 (Asian lineage-derived American strain, Puerto Rico, 2015) which had been passaged on Vero cells (African green monkey kidney cells) and C6/36 cells.

**ZIKV and Autophagy Induction:**

Assessment of the ability of ZIKV to induce autophagy by immunoblot and confocal microscopy was performed as described in the main text as described for DENV-2.

**Autophagy-ZIKV Interactions:**

The effects of chemical modulators on ZIKV infection of Aag2 cells was performed as described in the main text for DENV-2.

**MTT Assay**

Aag-2 cells were maintained in minimal essential media (MEM) supplemented with 10% FBS, non-essential amino acids, L-glutamine, anti-biotic/anti-mycotic and sodium bicarbonate. Cells were seeded (1x10^5^ cells/ well) in a Nunc Microwell 96-Well Optical-Bottom Plate and allowed to grow at 28°C until confluent. Once confluent, MEM was replaced with 200 µL fresh media and cells treated with either 1 µM Bafilomycin, 10 µM Spautin, 1 µM Torin, 1% DMSO or left untreated (n=4), plates were then placed at 28°C for 24 hours. After treatment the cells were tested for viability using the CyQUANT MTT Cell Viability Assay kit (Invitrogen). MEM was replaced with 100 µL fresh media, then treated with 10 µL of 12 mM MTT in PBS and incubated at 37°C for 4 hours. Following the incubation, 100 µL of SDS-HCl solution was added, mixed, and then incubated for an additional 4 hours at 37°C. The plate was then removed and the absorbance measured at 570 nm using the Endpoint setting on the Synergy H1 Hybrid multi-mode Microplate Reader (BioTek).
