# Supplementary figures and images for: The impact of autophagy on arbovirus infection of mosquito cells
Source: PLoS Negl Trop Dis. 2020 May 18;14(5):e0007754. doi: 10.1371/journal.pntd.0007754 (PMC7259790; doi:10.1371/journal.pntd.0007754)

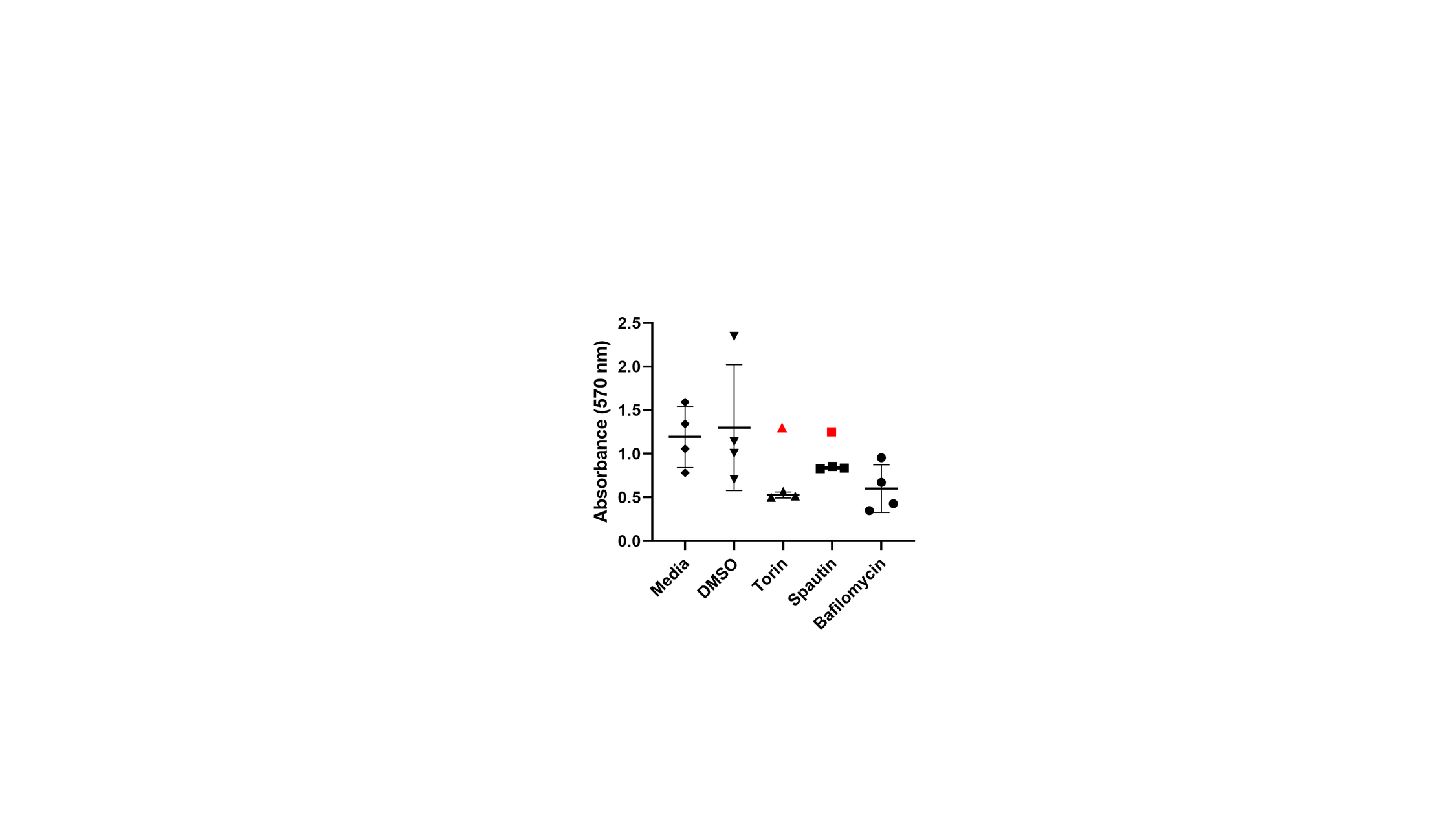

Supplement: S1 Fig — Aag2 cells were treated with either torin-1 (1 μM), spautin-1 (10 μM), bafilomycin A1 (1 μM), 1% DMSO, or untreated for 24 hrs (n = 4). Subsequently, the MTT cell viability assay was performed to determine if the chemicals had cytotoxic effects. Data was analyzed by one-way ANOVA with a Dunnett’s multiple comparisons test. Red data points were determined to be outliers by Grubb’s test for outliers and were not included in the analysis. (TIF) [file pntd.0007754.s002.tif]

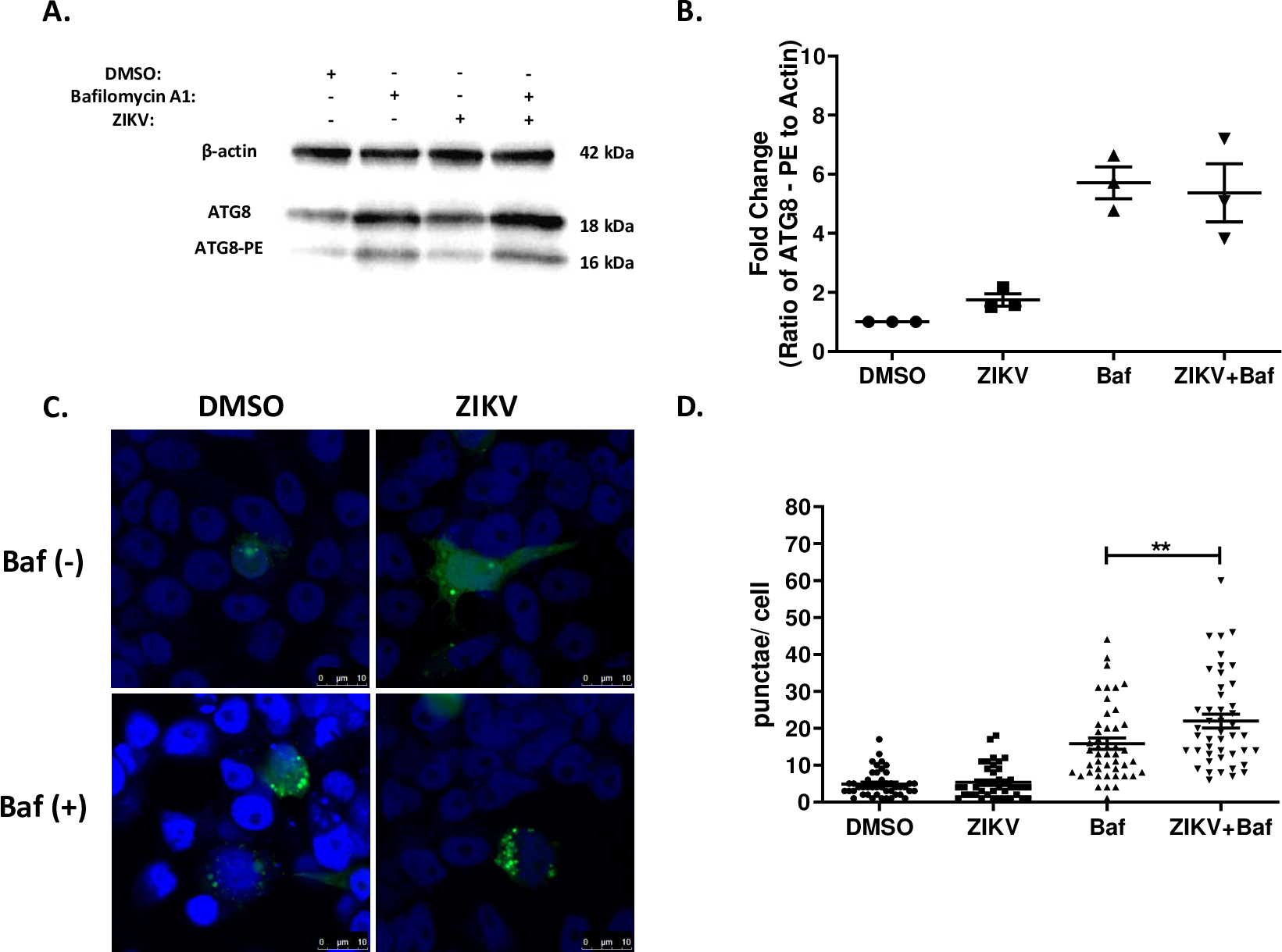

Supplement: S2 Fig — A) Representative immunoblot of Aag2 lysates upon ZIKV infection (M.O.I. 0.5) and/ or chemical treatment (1% DMSO, 1 μM bafilomycin A1, 1 μM torin-1 or 10 μM spautin-1) 48 hpi. B) Fold-change in the ratio of Atg8-PE to β-actin band intensities as determined by ImageJ. Includes data from four experimental replicates. C) Representative confocal microscopy images of Atg8-EGFP expressing Aag2 cells ± ZIKV infection (M.O.I. 0.1) and ±1 μM bafilomycin A1 24 hpi. Blue (nuclei), green (Atg8-EGFP + puncta). D) The number of Atg8+ puncta were quantified using the ImageJ Puncta Analyzer plug-in from ~50 Atg8-EGFP expressing Aag2 cells ± ZIKV infection (M.O.I. 0.1) and ±1 μM bafilomycin-A1 24 hpi. Combined data from three blinded experimental replicates. Data were analyzed by One-way ANOVA with a Sidak’s multiple comparisons test. (*) p<0.05, (**) p<0.01, (***) p<0.001. (TIF) [file pntd.0007754.s003.tif]

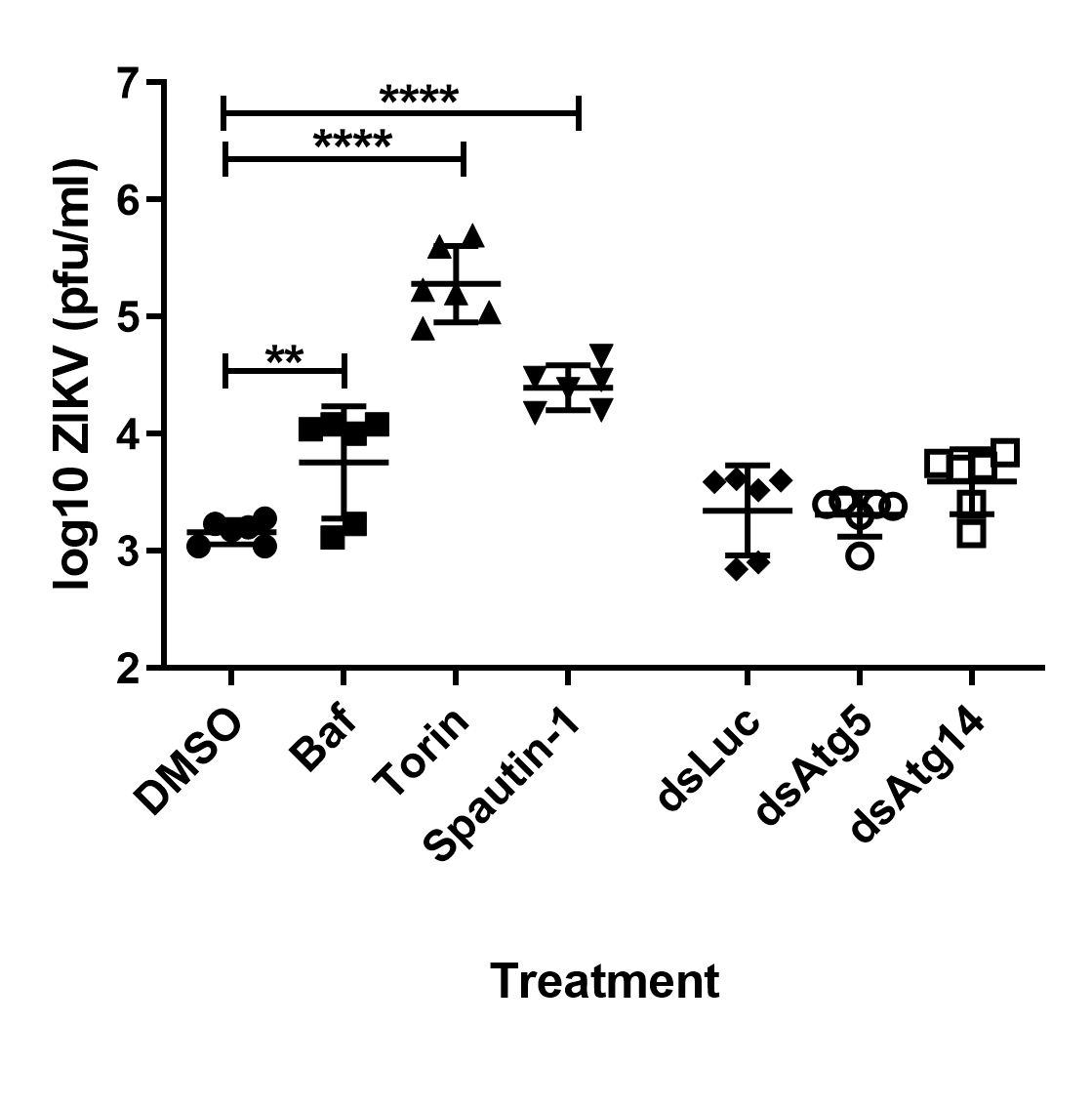

Supplement: S3 Fig — Aedes aegypti Aag2 cells were infected with ZIKV followed by chemical treatment (1% DMSO, 1 μM bafilomycin A1, 1 μM torin-1 or 10 μM spautin-1) or treated with dsRNA against Atg5, Atg14, or non-specific control luciferase genes two days prior to infection with ZIKV. Samples were collected for titration 48 hpi. Data was analyzed by one-way ANOVA with a Dunnett’s multiple comparisons test. (*) p<0.05, (**) p<0.01, (***) p<0.001. (TIF) [file pntd.0007754.s004.tif]

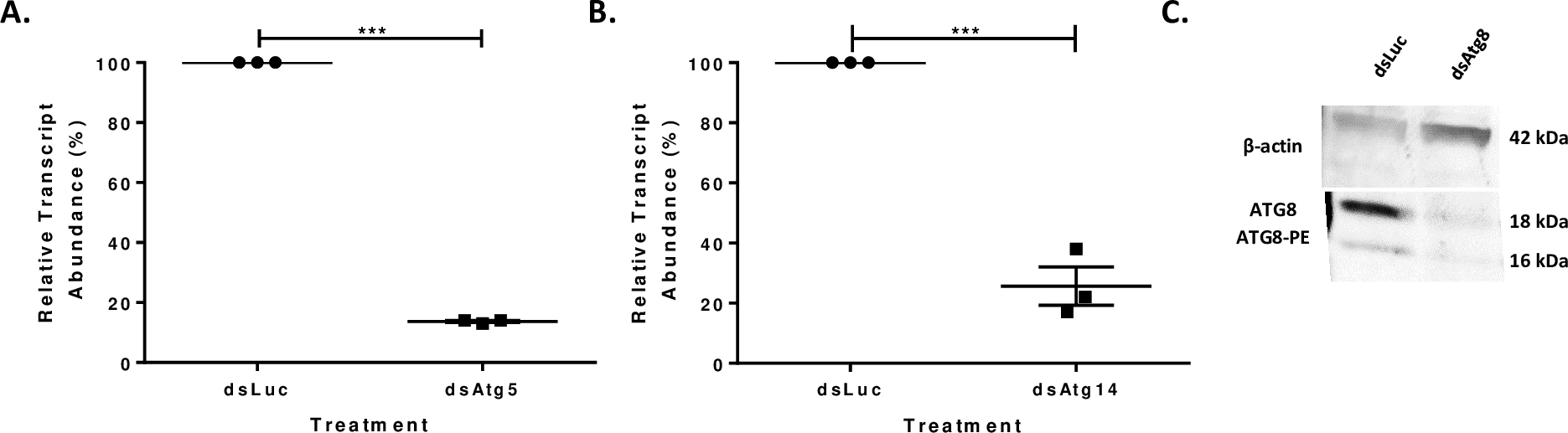

Supplement: S4 Fig — Aag2 cells were treated with dsRNA targeting Atg5, Atg14, or Atg8 and assayed for suppression 48 hours post transfection. Silencing efficiency of A) Atg5 and B) Atg14 was determined by ΔΔCT analysis with luciferase samples as the non-targeting control group and GAPDH as a reference gene. Data was analyzed with a two-tailed t-test. C) Silencing efficiency of Atg8 was determined by immunoblot. (TIF) [file pntd.0007754.s005.tif]
