# Supplementary material for: The impact of autophagy on arbovirus infection of mosquito cells
Source: PLoS Negl Trop Dis. 2020 May 18;14(5):e0007754. doi: 10.1371/journal.pntd.0007754 (PMC7259790; doi:10.1371/journal.pntd.0007754)
Supplement: Attachment — Submitted filename: PNTD-D-19-01476_reviewer comments.docx [file pntd.0007754.s006.docx]

**General Comments**

- Please make sure the numbering is continuous and does not re-start on each page. I’ve included the page numbers below for reference
- In the introduction there needs to be a few more statements making it clear when you are referring to humans or the invertebrate vector (when referring to autophagy responses)
- This is a cell line study not an *in vivo* study, please make that denotation clear throughout the paper
- Add in serotype specific information about DENV when referring to the virus in the body of the paper
- I think the use of a general autophagy pathway figure would increase understanding of the pathway and modulators that you are presenting
- Please include the timing of the experiment in the abstract and introduction and mention these timepoints more throughout – it seems to be important for your story but absent in your discussion (24 or 48 hours post chemical modulators)
- Be consistent with naming – spautin 1 sometimes includes a hyphen and sometime does not
- Many statements in the result are unfounded or speculative. Please remove the speculation/explanatory statements into the discussion and provide references for other statements (i.e. C6/36 lack of RNAi pathway to DENV, viral replication rate differences, autophagic flux, etc.)
- The results section (as currently written) needs work to explain why all experiments were not fully factorial in design. This is not clear and is confusing to the reader.
- There is very little mention of the autophagy/apoptosis work completed *in vivo* in mosquitoes – most of the comparisons are between mammalian cell lines. Please include more comparisons between the study organism of interest.

**Specific Comments**

Page 2 Line 16: no evidence of ‘limited’ role given yet – maybe an unknown role.

Page 3 Line 2: italicize *Aedes aegypti*

Page 3 Line 9: change to within cell lines from vector mosquitoes

Page 5 Line 13: change to “As a result we know very little about the role of autophagy during arbovirus infection within the invertebrate vector.”

Page 5 Line 23: take out ‘cells’

Page 5 Line 23: the spautin-1 text is not seemingly related to the rest of the paragraph. Please place elsewhere

Page 6 Line 2: I don’t think with the current dataset the word ‘limited’ is the correct term – what you have presented is interesting and changes titers – please use a better work for this (unknown, etc.)

Page 6 Line 12: This is the first mention of this study using mammalian cell lines. Mention this earlier.

Page 6 Line 19: Insert comma after families

Page 7 Line 5: Insert hyphen in torin-1

Page 7 Lines 4-11: Define spautin 1’s entire name (specific and potent autophagy inhibitor-1). It would also be helpful here to summarize each modulator’s overall effect on autophagy (i.e. torin-1 induces, spautin 1 and bafilomycin A1 inhibit). This is the first mention of bafilomycin A1 – please mention this earlier.

Page 7 Line 14: Change to “…cells were treated with 1% DMSO, and 1 μM of either bafilomycin A1, or torin-1 or 10 μM of spautin-1.”

Page 8 Line 4: Reference ImageJ

Page 8 Line 8: remove hyphen from spautin 1

Page 8 Line 10: Why were the CHIKV samples harvested at a separate time point?

Page 8 Line 19: Change to ‘were seeded’

Page 9 Lines 9-10: Why was silencing efficiency determined differently for different genes?

Page 9 Line 17: remove hyphen from spautin 1

Page 9 Line 18: What happened to the dsAtg8 treatment?

Page 9 Line 19: Why only DENV here?

Page 10 Line 1: Please reference C6/36 having a dysfunctional RNA interference pathway with DENV

Page 10 Line 3: remove hyphen from spautin 1

Page 10 Line 4: Why not at 24h as well?

Page 11 Lines 9-12: Include what program was used to complete statistics and the level of significance

Page 12 Line 1: you used an inducer and an inhibitor and saw an effect? Did you mean spautin 1 and bafilomycin A1 (both inhibitors)?

Page 12 Lines 3-4: I don’t think this evidence alone shows that the modulators are functioning as expected – none of the modulators worked on their own.

Page 12 Lines 10-Page 13 Line 9: Why is bafilomycin A1 only included here? What happened to torin and spautin?

Page 12 Lines 15 – 16: Add this into the discussion and remove from the results section.

Page 12 Line 16: Remove the word ‘consequently’

Page 13 Lines 3-4: What is the rate of virus replication in these cells?

Page 13 Lines 7-9: This belongs in the discussion.

Page 13 Line 12-Page 14 Line 12: Please make separate paragraphs for each study. What happened to ZIKV in the dsAtg 5, 14, and 8 treatments?

Page 14 Line 15 – Page 15 Line 3: The reason for only including these cell lines in only part of the study is unclear and seems superfluous.

Page 15 Line 17: Is it induced? You say otherwise on Page 12 Lines 11-13.

Page 16 Line 1: What is the control here?

Page 16 Line 1: Mention what ‘pr’ is before discussing results.

Page 16 Line 11: When is ‘pr’ tested in this paper? Or are these unpublished results?

Page 15 Line 20 – Page 17 Line3: Please put the 2^nd^ half of this paragraph up front so the reader understands why we are talking about viral maturation – this isn’t clear until the end of the paragraph.

Page 17 Lines 4-5: Only at specific times for some viruses – please mention this.

Page 17 Line 8: Could it also be that those autophagy related proteins are important in other systems but other Atg proteins are important in Aag2 cells or even that Aag2 cells don’t have a replicable autophagy system to an *in vivo* model?

Page 17 Lines 19-20: Is there any genomic evidence for this?

Page 17 Line 21: And bafilomycin A1 treatment

Page 18 Line 1: Change to ‘Aag2 cells’

Page 18 Lines 1-4: This could also be because the cell lines are derived from different mosquito stages (Aag2 is from embryos while C6/36 is from larvae)

Page 18: Line 5: This is only one method. Embryos, larvae, ovaries and adults have been used to create cell lines before

Page 18 Line 19: Cite RNAi genes not significantly upregulated upon infection please

Page 19 Lines 1-17: No mention of bafilomycin?

Table 1: Merge the cells referring to the same gene and include the accession # column beside the gene

Figures 1-3: The writing on these figures is not legible – please fix this and make sure the scale bars on the images are visible.

Figures 6-7: Please include the virus on the y-axis
