# Supplementary material for: The impact of autophagy on arbovirus infection of mosquito cells
Source: PLoS Negl Trop Dis. 2020 May 18;14(5):e0007754. doi: 10.1371/journal.pntd.0007754 (PMC7259790; doi:10.1371/journal.pntd.0007754)
Supplement: Attachment — Submitted filename: Brackney Review.docx [file pntd.0007754.s007.docx]

A limited role for autophagy during arbovirus infection of mosquito cells

Brackney & Correa

**General remarks**

In this manuscript, the authors investigate the role of autophagy upon arbovirus infection in mosquito cells. It is a relevant topic that is of interest for the field and that remains so far understudied. In this manuscript, the authors make use of mosquito cell lines (Aag2 mostly) to investigate whether autophagy is induced by arboviruses. In addition, they make use of well-known inhibitors of autophagy to test its impact on viral replication. Finally, they also knock-down autophagy-related genes and test whether this has an impact on virus replication.

Overall, the results show that autophagy, in mosquito cell lines, does not seem to play a major role in arbovirus control, and I appreciate that these conclusions are written clearly and explicitly. Maybe this was not originally expected, given the higher importance of autophagy as an antiviral pathway in mammalian cell lines, but nevertheless is an interesting finding that I believe, once revised, deserves publication.

However, some major questions need to be addressed, in particular regarding cell viability. In addition, the manuscript need further improvements and polishing: some figures annotations are incomplete, abbreviations not defined, and definition of technical terms not given. In addition, it would have been useful to have numbering throughout the text, and not starting at 1 for each page.

**Major remarks**

1) It is common practice when using compounds interfering with essential cellular functions to test different dosages, and estimate their impact on cell viability, given that could potentially impact experimental results and conclusions. Aag2 cells are well-known to be sensitive to stresses, changes in media composition or pH, and easily collapse.

Have different drug dosages been tested for Bafilomycin A1, torin-1 or spautin-1 on Aag2 cells? How did their concentration impact cell viability? How were the concentrations used in this manuscript chosen?

Ideally, one would choose a dosage at which autophagy function is altered without compromising cellular viability, which could lead to confounding results.

Such assays should be included in the manuscript as they might impact findings and conclusions.

2) In figure S2C, when comparing the “dsLuc” and “dsLuc + DENV” levels, it is noticeable that DENV infection reduces Atg8 levels, while Atg8-PE levels remain stable. This is not consistent with the immunoblot in figure 1, in which DENV infection alone does not impact Atg8 levels. What explains this discrepancy? The effect of DENV infection alone on Atg8 levels should be consistent in the manuscript, as the reader is for now left to wonder which result is representative. More immunoblots should be performed with DENV, and eventually with CHICKV and ZIKA, at several time points post-infection.

**Minor remarks**

1) This applies to all immunoblots: For each blot, please add the approximate size (in kDa) of the protein in front of each band, and show them by decreasing size. Actin is sometimes shown above Atg8 (Fig S2), sometimes below (Fig 1). This needs to be consistent, and coherent with protein weight, as is custom for such figures.

2) The “-PE” in Atg8-PE has not been defined in the manuscript, and I don’t think it’s reasonable to expect every reader to know what “-PE” means.

3) Line 3, page 8: Please define “Autophagic flux” and clarify that point. How do you come to the conclusion that Aag2 cells have high autophagic flux? And what is it? This claim seems unsupported and, if not justified better, should be removed from the paragraph, and from the paragraph below where it is mentioned again.

4) Line 18, page 8: if the result is not significant, it should not be stated that an increase is observed.

5) Fig 6 and 7. Please write which virus is used on the y-axis label, as done on previous figures.

6) When describing Figure 4 in the text, please name each panel “4A”, “4B”, …

7) DENV is alternatively written “DENV” or “DENV-2”, please be precise and consistent.

8) The DAPI coloring appears alternatively in blue or purple, please be consistent in coloring as this is confusing for the reader. It is also not clear when looking at the immunofluorescence pictures in Fig 1C, 2C and 3C what the purple/blue and green colors represent. Please add a more explicit legend, in addition to mentioning the treatments.

9) It would be good to mention the time points at which samples were harvested in the figure legends.
