# Supplementary material for: The impact of autophagy on arbovirus infection of mosquito cells
Source: PLoS Negl Trop Dis. 2020 May 18;14(5):e0007754. doi: 10.1371/journal.pntd.0007754 (PMC7259790; doi:10.1371/journal.pntd.0007754)
Supplement: Attachment — Submitted filename: Response to Reviewers.docx [file pntd.0007754.s008.docx]

Response to Reviewers:

We would like to thank the reviewers first and foremost for taking the time to thoroughly review our manuscript and provide constructive critiques. Overall the reviews were positive, but there were also many concerns and suggested improvements. Below we have provided a detailed response to each of the reviewer’s comments.

Reviewer 1:

1. “In addition, it would have been useful to have numbering throughout the text, and not starting at 1 for each page.” This change has been made.

2. The reviewer suggested that we provide toxicity data in mosquito cells for each of the chemicals used because if toxicity exists it could obfuscate our results. We have provided a new supplemental figure showing results from a MTT cell viability assay as a test of toxicity for all three chemicals (Torin 1, Spautin 1 and Bafilomycin A1) at the concentrations used throughout the manuscript in Aag2 cells.

3. “In figure S2C, when comparing the “dsLuc” and “dsLuc + DENV” levels, it is noticeable that DENV infection reduces Atg8 levels, while Atg8-PE levels remain stable. This is not consistent with the immunoblot in figure 1, in which DENV infection alone does not impact Atg8 levels. What explains this discrepancy? The effect of DENV infection alone on Atg8 levels should be consistent in the manuscript, as the reader is for now left to wonder which result is representative. More immunoblots should be performed with DENV, and eventually with CHICKV and ZIKA, at several time points post-infection.” We agree that there appears to be a difference in the steady state levels of Atg8 between dsLuc and dsLuc+DENV; however, the more important issues are that 1) Atg8-PE is not altered relative to actin and 2) that we achieved the desired suppression. This discrepancy probably arises from the inherent variability associated with analyzing band intensities in western blots. For instance, if the reviewers look at Figure 1b, the fold change in the ratio of Atg8-PE to actin, is not always consistent between experimental replicates (this data is a quantification of the western blots). Of the four replicates in the DENV+Baf group one is clearly less than the other. This is science. Regardless, this inconsistency doesn’t change the conclusion that we can effectively suppress Atg8 using dsRNA. To avoid confusion and because it is not necessary to demonstrate suppression we will remove the lanes dsLuc+DENV and dsAtg8+DENV from the figure. It is unclear to us the benefit that additional immunoblots would provide considering that we have presented data from 4 (Fig 2b), 3 (FigS2b) and 3 (Fig 3b) immunoblots of DENV, ZIKV and CHIKV, respectively.

4. “This applies to all immunoblots: For each blot, please add the approximate size (in kDa) of the protein in front of each band, and show them by decreasing size. Actin is sometimes shown above Atg8 (Fig S2), sometimes below (Fig 1). This needs to be consistent, and coherent with protein weight, as is custom for such figures.” The suggested changes have been made.

5. “The “-PE” in Atg8-PE has not been defined in the manuscript, and I don’t think it’s reasonable to expect every reader to know what “-PE” means.” The term “phosphatidyethanolamine” has been added to the first reference of its use in the manuscript (pg5 ln 106).

6. Line 3, page 8: Please define “Autophagic flux” and clarify that point. How do you come to the conclusion that Aag2 cells have high autophagic flux? And what is it? This claim seems unsupported and, if not justified better, should be removed from the paragraph, and from the paragraph below where it is mentioned again. We have added the following text at the first mention of the term autophagic flux: “autophagic flux (the rate of autophagosomal turnover)”. We disagree with the reviewer’s assessment that our conclusion that the rate of autophagic flux is high in Aag2 cells is not justified. Bafilomycin A1 blocks autophagosome fusion with and the ultimate acidification of lysosomes. If there is a high rate of steady state autophagic turnover than it may be difficult to observe differences in autophagy induction because the autophagosomes are rapidly degraded; however, the addition of bafilomycin A1 prevents this degradation from occurring thereby allowing the accumulation of autophagosomes and their ultimate quantification. This is what we observed for DENV, ZIKV and CHIKV. As such, we feel confident in our conclusion as they are currently written.

7. “Line 18, page 8: if the result is not significant, it should not be stated that an increase is observed.” We believe it is worth noting that aside from one “outlier” replicate, the data demonstrates an increase in Atg8-PE accumulation upon CHIKV infection. This is further supported by the confocal microscopy. Because we state that the finding is not significant, we do not feel it is necessary to change the text.

8. “Fig 6 and 7. Please write which virus is used on the y-axis label, as done on previous figures.” The suggested changes have been made.

9. “When describing Figure 4 in the text, please name each panel “4A”, “4B”, … “ The suggested changes have been made.

10. “DENV is alternatively written “DENV” or “DENV-2”, please be precise and consistent.” All references to our specific work have been changed to DENV-2. In other instances when referring to dengue virus in general the form DENV has been unchanged.

11. “The DAPI coloring appears alternatively in blue or purple, please be consistent in coloring as this is confusing for the reader. It is also not clear when looking at the immunofluorescence pictures in Fig 1C, 2C and 3C what the purple/blue and green colors represent. Please add a more explicit legend, in addition to mentioning the treatments.” For consistency throughout the manuscript all nuclei have been changed to blue. Additional legend text has been added for clarity.

12. “It would be good to mention the time points at which samples were harvested in the figure legends.” We double-checked and this information is already present in each legend figure.

Reviewer 2:

- Please make sure the numbering is continuous and does not re-start on each page. I’ve included the page numbers below for reference. Changed as suggested.
- In the introduction there needs to be a few more statements making it clear when you are referring to humans or the invertebrate vector (when referring to autophagy responses) Having re-read the introduction a number of times we feel that the way that it is currently written clearly highlights the differences between what we know from mammalian systems (basically everything) and that which is known from invertebrate systems (basically nothing). Therefore we have not altered the text.
- This is a cell line study not an *in vivo* study, please make that denotation clear throughout the paper We think that the reviewers primary concern was with the last two sentences of the author summary. We have made changes to make it clear that this study was not performed in mosquitoes, but rather mosquito cell culture.
- Add in serotype specific information about DENV when referring to the virus in the body of the paper Changes were made as suggested.
- I think the use of a general autophagy pathway figure would increase understanding of the pathway and modulators that you are presenting. We agree that this could be helpful, but considering that this is an online journal it is probably safe to assume that the reader has access to the internet and could quickly query autophagy pathway diagrams for their reference if need be. For these reasons we don’t feel the addition of an autophagy pathway would substantially strengthen the manuscript.
- Please include the timing of the experiment in the abstract and introduction and mention these timepoints more throughout – it seems to be important for your story but absent in your discussion (24 or 48 hours post chemical modulators). In the manuscripts original version, time points were included in all of the figure legends, results and materials and methods section. As with any autophagy experiment we agree that timing is important; however, our presentation of the chosen time points is consistent with other published autophagy studies. It is our opinion that littering the text with time point references does not change the conclusions and would detract from the readability of the manuscript. Therefore, changes were not made.
- Be consistent with naming – spautin 1 sometimes includes a hyphen and sometime does not Changes were made as suggested.
- Many statements in the result are unfounded or speculative. Please remove the speculation/explanatory statements into the discussion and provide references for other statements (i.e. C6/36 lack of RNAi pathway to DENV, viral replication rate differences, autophagic flux, etc.). We agree there were some instances where we provided too much explanation in the results sections and as a result some of those instances have been moved to the discussion or materials sections. Conversely, there were some instances where explanation/ interpretation was necessary to contextualize the results and the flow of the experimentation to the reader (e.g. interpreting our autophagic flux data). In these instances the explanatory statements were left in the results.
- The results section (as currently written) needs work to explain why all experiments were not fully factorial in design. This is not clear and is confusing to the reader. The incongruity in our data stems from our analysis of ZIKV. The simple explanation is that ZIKV was tacked on at the end to see if the results were consistent across flaviviruses and because, at the time of the experiments, ZIKV was big in the headlines. Because the data set for ZIKV is not as complete as that of DENV and CHIKV and it doesn’t change our conclusions we have decided to move the ZIKV data to supplemental. Consequently, portions throughout the manuscript have been modified to reflect this change.
- There is very little mention of the autophagy/apoptosis work completed *in vivo* in mosquitoes – most of the comparisons are between mammalian cell lines. Please include more comparisons between the study organisms of interest. To our knowledge only two papers have examined autophagy in adult mosquitoes. One focuses on the role of autophagy during oogenesis, which was cited, and another showing an increase in autophagy following a bloodmeal and that there is a correlation between autophagy activation and autophagy induction (not cited). The first was cited to demonstrate that autophagy is intact and fully functional in mosquitoes. The second paper will be added as a reference to further emphasize this point; however, we did not specifically discuss these papers in detail because neither really examined autophagy in the context of a virus infection. Yes the second paper examined autophagy by immunoblot in midguts following DENV infection, but don’t really examine this in detail. Rather they just demonstrate that Caspase 7 seems to regulate autophagy activity. Consequently there is not much to discuss with regards to virus/ autophagy interactions which are the central theme of this manuscript. Therefore changes have not been made.

**Specific Comments**

Page 2 Line 16: no evidence of ‘limited’ role given yet – maybe an unknown role. Any reference to the term “limited” has been changed throughout the manuscript including the title.

Page 3 Line 2: italicize *Aedes aegypti* Done

Page 3 Line 9: change to within cell lines from vector mosquitoes Changed to “mosquito cells”

Page 5 Line 13: change to “As a result we know very little about the role of autophagy during arbovirus infection within the invertebrate vector.” Changed

Page 5 Line 23: take out ‘cells’ Changed

Page 5 Line 23: the spautin-1 text is not seemingly related to the rest of the paragraph. Please place elsewhere We respectively disagree and have thus not made this change.

Page 6 Line 2: I don’t think with the current dataset the word ‘limited’ is the correct term – what you have presented is interesting and changes titers – please use a better work for this (unknown, etc.) See above.

Page 6 Line 12: This is the first mention of this study using mammalian cell lines. Mention this earlier. It is mentioned in the final paragraph of the Introduction as well.

Page 6 Line 19: Insert comma after families Done

Page 7 Line 5: Insert hyphen in torin-1Done

Page 7 Lines 4-11: Define spautin 1’s entire name (specific and potent autophagy inhibitor-1). It would also be helpful here to summarize each modulator’s overall effect on autophagy (i.e. torin-1 induces, spautin 1 and bafilomycin A1 inhibit). This is the first mention of bafilomycin A1 – please mention this earlier. We have included the entire name for spautin. The original text already provides a brief description of each chemicals effect on autophagy. It states “torin-1, an autophagy inducer that works by inhibiting mTOR kinase, and spautin-1 (specific and potent autophagy inhibitor 1) (Tocris Bioscience), which inhibits autophagy by interfering with USP10 and USP13, two ubiquitin-specific peptidases, resulting in the degradation of class III phosphatidylinositol-3 kinase complexes (23). Bafilomycin A1 (InvivoGen), an inhibitor of V-ATPase”.

Page 7 Line 14: Change to “…cells were treated with 1% DMSO, and 1 μM of either bafilomycin A1, or torin-1 or 10 μM of spautin-1.” Changing this would be incorrect. The 1% DMSO treatment was a separate control treatment. It was not added to each well in addition to DMSO. Granted the chemicals were resuspended in DMSO, but this is made clear in the M&M.

Page 8 Line 4: Reference ImageJ Done

Page 8 Line 8: remove hyphen from spautin 1Done

Page 8 Line 10: Why were the CHIKV samples harvested at a separate time point? Because they replicate faster than either DENV or ZIKV. That is correct.

Page 8 Line 19: Change to ‘were seeded’ Done

Page 9 Lines 9-10: Why was silencing efficiency determined differently for different genes? We only had an antibody for Atg8, therefore RT-qPCR was used to determine gene silencing efficiency for the other two genes.

Page 9 Line 17: remove hyphen from spautin 1Done

Page 9 Line 18: What happened to the dsAtg8 treatment? Accidently left out; has been added.

Page 9 Line 19: Why only DENV here? Accidently left out CHIKV; it has been added.

Page 10 Line 1: Please reference C6/36 having a dysfunctional RNA interference pathway with DENV Citation added.

Page 10 Line 3: remove hyphen from spautin 1Done

Page 10 Line 4: Why not at 24h as well? Earlier studies in the manuscript demonstrated no significant difference in outcomes between 24 and 48 in Aag2 cells so we chose a single time point.

Page 11 Lines 9-12: Include what program was used to complete statistics and the level of significance Done

Page 12 Line 1: you used an inducer and an inhibitor and saw an effect? Did you mean spautin 1 and bafilomycin A1 (both inhibitors)? There was no effect observed for any of the chemicals alone; however, when the inducer Torin 1 was combined with baf there was an increase in Atg8-PE. Because spautin 1 did not result in detectable levels of inhibition on its own we induced early autophagy with Torin 1 and blocked late autophagy with baf so that we could detect this induction. Spautin 1 addition should counteract the effects of torin 1 therefore we should see a reduction in the accumulation of Atg8-PE in the group exposed to spautin, torin and baf compared to the group just containing torin and baf. This is indeed what we observed.

Page 12 Lines 3-4: I don’t think this evidence alone shows that the modulators are functioning as expected – none of the modulators worked on their own. This is precisely why we come to the conclusion that autophagic flux is high in Aag2 cells. In instances of high autophagic flux (the steady state rate of autophagosomal turnover/ degradation whereby Atg8-PE signal is lost) it is commonplace to use bafilomycin A1 because it basically prevents the autophagosomes containing the Atg8-PE marker from being degraded by lysosomes and the loss of Atg8-PE signal. The addition of baf is to allow for the accumulation of Atg8-PE + autophagosomes so that they can be detected. Torin 1 addition by itself should result in the generation of more Atg8-PE + autophagosomes; however, if they are being degraded quickly we won’t observe that accumulation without preventing downstream degradation.

Page 12 Lines 10-Page 13 Line 9: Why is bafilomycin A1 only included here? What happened to torin and spautin? Because we are specifically looking at the effects of the virus on autophagy induction. As above, if the virus were to induce autophagy in an environment with high autophagic flux we wouldn’t be able to detect it. Therefore, the addition of baf is to slow down the degradation allowing us the opportunity to see if the virus(es) is actually inducing autophagy.

Page 12 Lines 15 – 16: Add this into the discussion and remove from the results section. We feel it is necessary to leave as is for interpretation purposes.

Page 12 Line 16: Remove the word ‘consequently’ This was left in because the last sentence was left in place.

Page 13 Lines 3-4: What is the rate of virus replication in these cells? It is unclear what the reviewer is asking. Typically, alphaviruses, such as CHIKV, replicate faster than flaviviruses hence our focus on earlier time points (i.e. 24 hpi) for CHIKV and later time points (i.e. 48 hpi) for DENV.

Page 13 Lines 7-9: This belongs in the discussion. We respectfully disagree. Some interpretation of the data is needed in the results section to help contextualize the results.

Page 13 Line 12-Page 14 Line 12: Please make separate paragraphs for each study. What happened to ZIKV in the dsAtg 5, 14, and 8 treatments? ZIKV has been moved to supplemental (see above). Other suggested changes are stylistic and we don’t feel making this change is necessary. The idea was to put all data relevant to induction/ inhibition whether it be dsRNA suppression or chemicals into one section.

Page 14 Line 15 – Page 15 Line 3: The reason for only including these cell lines in only part of the study is unclear and seems superfluous. We respectfully disagree. The BHK-21 cells were used because there is another report showing the use of DENV-2 and spautin 1 in BHK cells. We included the BHK to demonstrate that our use of spautin 1 is consistent with the literature. We used C6/36 cells because they represent another commonly used mosquito cell line. This was done for comparisons and to demonstrate that use of chemicals in mosquito cell line might have different effects in another.

Page 15 Line 17: Is it induced? You say otherwise on Page 12 Lines 11-13. Actually we say that torin 1 does induce, but we can’t detect it without bafilomycin A1.

Page 16 Line 1: What is the control here? The text “non-spautin 1 treated cells” has been added

Page 16 Line 1: Mention what ‘pr’ is before discussing results. We explain pr in the subsequent sentence. As such the text remains unchanged.

Page 16 Line 11: When is ‘pr’ tested in this paper? Or are these unpublished results? Pr retention on the virion was not tested in this paper. The purpose of the discussion section is to contextualize ones results in the larger body of literature. That is the point of us discussing pr and how our results fit in with the findings from other papers.

Page 15 Line 20 – Page 17 Line3: Please put the 2^nd^ half of this paragraph up front so the reader understands why we are talking about viral maturation – this isn’t clear until the end of the paragraph. This is a stylistic request and we respectfully disagree. Changes have not been made.

Page 17 Lines 4-5: Only at specific times for some viruses – please mention this. We have included the text “at the assayed time points” to the end of the sentence.

Page 17 Line 8: Could it also be that those autophagy related proteins are important in other systems but other Atg proteins are important in Aag2 cells or even that Aag2 cells don’t have a replicable autophagy system to an *in vivo* model? Autophagy is highly conserved from yeast to humans so it is highly unlikely that the autophagy functions differently in mosquito cells. Further it would be extremely unlikely that proteins in highly conserved pathways would differ significantly in function between mosquitoes and their derived cell lines. As with the antiviral RNAi, it is possible that Aag2 cells have a dysfunctional autophagy pathway; however, our results would suggest otherwise and the loss of autophagy would be lethal to the cells. Therefore we feel that this is an improbable explanation.

Page 17 Lines 19-20: Is there any genomic evidence for this? No. This is a discussion where we should be able to speculate within reason.

Page 17 Line 21: And bafilomycin A1 treatment Bafilomycin was not tested in the C6/36 cells so we didn’t bring it up in the context of the discussion about C6/36 cells. While bafilomycin is technically considered an autophagy inhibitor, it is also functions late in the pathway and is commonly used to study autophagic flux. Therefore, it was not tested in C6/36 cells because we were particularly interested in the effects of autophagy from the beginning of the pathway. Both spautin and torin act early in the pathway and, thus were chosen for the comparison.

Page 18 Line 1: Change to ‘Aag2 cells’ Done

Page 18 Lines 1-4: This could also be because the cell lines are derived from different mosquito stages (Aag2 is from embryos while C6/36 is from larvae) Good point, extra text has been added.

Page 18: Line 5: This is only one method. Embryos, larvae, ovaries and adults have been used to create cell lines before See above.

Page 18 Line 19: Cite RNAi genes not significantly upregulated upon infection please The citation is present at the end of the sentence.

Page 19 Lines 1-17: No mention of bafilomycin? Again, baf is a late stage inhibitor and is commonly used to assess autophagic flux. We are specifically discussing the early events that spautin and torin inhibit or induce, respectively.

Table 1: Merge the cells referring to the same gene and include the accession # column beside the gene Done

Figures 1-3: The writing on these figures is not legible – please fix this and make sure the scale bars on the images are visible. Done

Figures 6-7: Please include the virus on the y-axis Done
