# Supplementary material for: The impact of autophagy on arbovirus infection of mosquito cells
Source: PLoS Negl Trop Dis. 2020 May 18;14(5):e0007754. doi: 10.1371/journal.pntd.0007754 (PMC7259790; doi:10.1371/journal.pntd.0007754)
Supplement: Attachment — Submitted filename: PNTD-D-19-01476_R1_reviewer comments.docx [file pntd.0007754.s009.docx]

**General Comments:**

- I think adding in some of the results from published RNAseq data (arbovirus infected mosquitoes) relating to your genes of interest could be interesting

- Why is there an increased concentration of B-actin in the immunoblot in the dsAtg8 treatment in Figure S4?

- Please avoid the use of terms like ‘marginal increase’, especially in comparison to results that were statistically significantly different. If the result was not significant, it should be left as so.

**Specific comments:**

- Line 84: add a comma after engulfed

- Line 118: remove the word in before DENV-2 titers

- Line 135: add the word using after carried out

- Line 138: What type of Vero cells?

- Line 151: why 10x more spautin-1?

- Line 167: concentration for bafilomycin A1 here? 1uM again?

- Line 240: Was a one-way ANOVA used to compare the MTT assay results?

- Line 299: Change resulting to resulted

- Line 300: add the word and after levels

- Line 401: Change insect-only viruses to insect-specific viruses

- Line 420: Add the word this before is

- Line 423: Change consistent to to consistent with

- Line 441: Add the word and after the comma

- Change Figure 2 legend to read DENV-2 as to match other figure legend titles

- Do not include non-significant difference labels on figures (presumably other groups that are not bracketed together were compared and were not significant)

- The labelling changes between Figure 1 and the other figures (legend is on top and ATG8 and ATG8-PE have changed weights)

- Could Figure 7 be split into two panels? One for Aag2 cells and one for C6/36 cells?

- Could Figure S3 be labelled with ds denotation rather than si as used for earlier figures?
