# Supplementary material for: The impact of autophagy on arbovirus infection of mosquito cells
Source: PLoS Negl Trop Dis. 2020 May 18;14(5):e0007754. doi: 10.1371/journal.pntd.0007754 (PMC7259790; doi:10.1371/journal.pntd.0007754)
Supplement: Attachment — Submitted filename: PNTD-D-19-01476_reviewer comments - 3.19.20.docx [file pntd.0007754.s011.docx]

**Specific Small Editorial Comments:**

- Line 86: Comma after recruitment events

- Line 120: Change highlight to highlights

- Line 130: Add space between brackets

- Line 203: Change and dsAtg14 to or dsAtg14

- Line 243: Ident start or paragraph

- Line 308: define hp or just use hours post

- Line 636: Includes data – does this mean data is representative or combined?

- Line 676: Outlier analysis via Grubb’s test is not in the methods – please add this in.

- Line 697: Change colon at the end of Figure S4 title to a period

- Line 698: Change and Atg8 to or Atg8

- Figure 5: Please keep data point shapes the same for each group between panel A and B

- Figure S3: Add in ZIKV to the y-axis title

- Notation for ‘times’ as x and X – please chose either lower or uppercase

- Notation for hours as h. and hrs. – please chose one

- Notation for multiplicity of infection as M.O.I. and MOI – please chose one

- Please add in paragraph indentations for the results section
