# Supplementary material for: The impact of autophagy on arbovirus infection of mosquito cells
Source: PLoS Negl Trop Dis. 2020 May 18;14(5):e0007754. doi: 10.1371/journal.pntd.0007754 (PMC7259790; doi:10.1371/journal.pntd.0007754)
Supplement: Attachment — Submitted filename: Reviewer Comments 2 Addressed.docx [file pntd.0007754.s010.docx]

Response to Reviewers:

We would like to thank the reviewers for taking the time to thoroughly review our manuscript and provide constructive critiques. Overall the reviews were positive with only minor corrections. We have hopefully addressed each of the concerns to the satisfaction of reviewers and editors. Below we have provided a detailed response to each of the reviewer’s comments.

Reviewer 2

- I think adding in some of the results from published RNAseq data (arbovirus infected mosquitoes) relating to your genes of interest could be interesting

We agree that this is an interesting question but feel that it is outside the scope of the manuscript. Nevertheless, we did perform a cursory analysis of five transcriptomics papers (Sim et al. 2013, Colpitt et al. 2011, Bonizzoni et al. 2012, Behura et al. 2011, and Zou et al. 2011). We searched the manuscripts and the available supplementary information using the search terms “autophagy” and “atg” and found no reference in the main text for either of these search terms. In one, Sim et al., five autophagy related genes were listed and identified in the supplemental materials; however, these genes were not significantly changed between DENV refractory and susceptible strains. Because there doesn’t seem to be much transcriptional differences in autophagy related genes upon infection we have not added additional text.

- Why is there an increased concentration of B-actin in the immunoblot in the dsAtg8 treatment in Figure S4?

Unfortunately, we do not know the answer to this question. It could be that our protein extractions were more efficient in the dsAtg8 group. We made an effort to load equal concentrations of protein to each lane of the western blot, but more could have been loaded to the dsAtg8 group or there could have been an uneven transfer of proteins at the upper M.W. where the actin bands arise. Aside from methodological artifacts we do not have a scientifically viable hypothesis as to why we observed increased actin levels in the dsAtg8 group.

- Please avoid the use of terms like ‘marginal increase’, especially in comparison to results that were statistically significantly different. If the result was not significant, it should be left as so.

In the Arbovirus Induced Autophagy section of the Results sections there were two instances that were changed to only reflect significant results. Instances of marginal or qualifying statements about statistical significance were removed.

**Specific comments:**

- Line 84: add a comma after engulfed Done

- Line 118: remove the word in before DENV-2 titers Done

- Line 135: add the word using after carried out Done

- Line 138: What type of Vero cells? Added Vero E6 (African green monkey kidney cells)

- Line 151: why 10x more spautin-1? This was the concentration used by the group that originally described Spautin-1 (Liu et al. 2011) as well as the group that assessed DENV infection of mammalian cells (Mateo et al. 2013). We thought it best to start with a concentration previously shown to work in the literature.

- Line 167: concentration for bafilomycin A1 here? 1uM again? Yes. This has been added.

- Line 240: Was a one-way ANOVA used to compare the MTT assay results? Yes. This information is now in the figure legend.

- Line 299: Change resulting to resulted Done

- Line 300: add the word and after levels Done

- Line 401: Change insect-only viruses to insect-specific viruses Done

- Line 420: Add the word this before is Done

- Line 423: Change consistent to to consistent with Done

- Line 441: Add the word and after the comma Done

- Change Figure 2 legend to read DENV-2 as to match other figure legend titles Completed. Also changed Figure 3 legend title to CHIKV to match other legend titles.

- Do not include non-significant difference labels on figures (presumably other groups that are not bracketed together were compared and were not significant) Non-significant brackets have been removed from figures as suggested.

- The labelling changes between Figure 1 and the other figures (legend is on top and ATG8 and ATG8-PE have changed weights) Figure 1 has been changed to match the other figures.

- Could Figure 7 be split into two panels? One for Aag2 cells and one for C6/36 cells? The two have been separated into two panels as suggested.

- Could Figure S3 be labelled with ds denotation rather than si as used for earlier figures? This error has been changed in Fig. S3.

Reviewer 3

-Most of the important points I raised have been addressed. However, I wish the authors would have been more open to the reviewers comments. They are meant to improve the manuscript and should be seriously considered, rather than answered lightly and with general comments such as "this is science".

It was not our intention to be flippant in our responses, and we apologize if we came off this way after our initial re-submission. We would also like to reiterate that we do very much appreciate the time and effort that each reviewer has spent providing suggestions for the improvement of our manuscript. The specific remark referenced was not necessary; however, the point we were trying to make was that biological systems have inherent variability and that sometimes there is not always a satisfactory explanation.

-I do not understand the reluctance to implement changes that where highlighted by both reviewers, such as a better description of time points throughout the manuscript, that overall stil lacks precision.

We have added better time point descriptions throughout the results section to provide more clarity.

-Adding a small scheme that recapitulates the autophagy pathway, and highlighting the steps that are affected by the various drugs used in the paper would be very welcome and does not necessitate much extra work. Explaining in a more comprehensive way what "autophagic flux" is in the manuscript, as has been done in the answers to reviewers would be helpful to readers.

We appreciate the suggestions, but we feel the manuscript clearly and succinctly present the status of arbovirus-autophagy research, states our research objectives, presents scientifically sound results, and a thorough interpretation. Having said that we have included a citation for an article that specifically addresses this very concern and includes a nice schematic.

-Line numbers were not always referred to properly in the reply to reviewers (where is Atg8-PE defined ? It is not page 5 line 106 as written) and leave a general impression of sloppiness.

Our apologies. We have made our best effort to make this new set of revisions easier to follow.
